# Optimization of Polyacrylic Acid Coating on Graphene Oxide-Functionalized Reverse-Osmosis Membrane Using UV Radiation through Response Surface Methodology

**DOI:** 10.3390/polym14183711

**Published:** 2022-09-06

**Authors:** Mohammad Yousaf Ashfaq, Mohammad A. Al-Ghouti

**Affiliations:** Environmental Science Program, Department of Biological and Environmental Sciences, College of Arts and Sciences, Qatar University, Doha 2713, Qatar

**Keywords:** polyacrylic acid, reverse osmosis, optimization, mineral scaling

## Abstract

Reverse osmosis (RO) is affected by multiple types of fouling such as biofouling, scaling, and organic fouling. Therefore, a multi-functional membrane capable of reducing more than one type of fouling is a need of the hour. The polyacrylic acid and graphene oxide (PAA-GO) nanocomposite functionalization of the RO membrane has shown its effectiveness against both mineral scaling and biofouling. In this research, the polyacrylic acid concentration and irradiation times were optimized for the PAA-GO-coated RO membrane using the response surface methodology (RSM) approach. The effect of these parameters on pure water permeability and salt rejection was investigated. The models were developed through the design of the experiment (DoE), which were further validated through the analysis of variance (ANOVA). The optimum conditions were found to be: 11.41 mg·L^−1^ (acrylic acid concentration) and 28.08 min (UV activation times) with the predicted results of 2.12 LMH·bar^−1^ and 98.5% NaCl rejection. The optimized membrane was prepared as per the model conditions, which showed an increase in both pure water permeability and salt rejection as compared to the control. The improvement in membrane surface smoothness and hydrophilicity for the optimized membrane also helped to inhibit mineral scaling by 98%.

## 1. Introduction

Reverse osmosis (RO) is a leading technology to produce clean water and cope with the rising demands of water resources. The technology offers various advantages such as high quality of treated water, environment-friendly process, and lesser energy consumption, but it also faces various obstacles in application due to membrane fouling. The feedwater contains a variety of foulants such as organic matter, microorganisms, and inorganic substances that settle on the membrane surface or clog the membrane pores, completely or partially compromising the membrane performance [1,2].

Membrane surface modification is one of the several methods used to develop fouling-resistant membranes [3,4]. While most of the previous research was aiming to develop membranes resistant to one of the fouling types such as biofouling or organic fouling, some researchers have recently demonstrated the need to develop multifunctional membranes capable of reducing different types of fouling. For this purpose, the use of composite materials such as the combination of an anti-biofouling agent and the anti-scaling agent has been tested. The use of graphene oxide (GO) to tackle biofouling on RO membranes is well-known in the literature [5,6]. Ashfaq et al. [7] combined the anti-bacterial properties of GO with polyacrylic acid (PAA), the latter for use as an antiscalant. The modified membrane showed its potential to combat scaling when it was tested using crossflow tests in the presence of calcium sulfate solution. To measure anti-biofouling performance, the bacteriostasis rates were determined, which showed that the membrane surface modification significantly reduced the bacterial growth rates. Similarly, another research group developed a PAA-tethered GO functionalized RO membrane in the presence of UV radiation, which was found to be capable of controlling both scaling and biofouling [8]. However, for the purpose of upscaling and industrial application, optimization of techniques for membrane surface modification is needed. The optimization procedure helps to fill various knowledge gaps related to the effect of concentration of coating material, processing times, and others. Therefore, in this research, the procedure for polymerization of PAA on GO functionalized RO membrane was optimized, and the response surface methodology (RSM) was used for this objective.

RSM is a commonly used optimization approach adopted to improve the quality of a process with significantly less cost consumption. It plays an important role in design application and process development. Through RSM, a statistical experimental design is developed that helps to optimize the membrane surface modification process with minimal experiments. With these experiments, the correlation between the identified parameters of the modification process and responses is determined [9,10]. With the help of RSM, the quadratic model is then developed to explain the relationships between parameters and responses. The analysis of variance (ANOVA) is conducted for model verification. From the established model, the most desirable and optimum parameters for a process can be accurately predicted, which are then experimentally tested to confirm the modeling results. Due to the benefits RSM offers, many researchers have applied it to optimize processes for membrane modification.

Chung et al., [11] used RSM to optimize the methacrylic acid concentration and UV activation time for surface modification of sulfonated-polysulfone membrane. It was found that 2.61 wt.% as the concentration of methacrylic acid, and 21.10 min as UV activation time were the optimized operating conditions that yielded 8.75 LMH·bar^−1^ pure water permeability and 95% rejection for humic acid. Similarly, Razali et al. [12] used RSM to optimize the membrane fabrication process for polysulfone and polyaniline composite membranes. The central composite design (CCD) of the RSM was employed and the optimized operating conditions were identified as 18.33 wt.% polyethersulfone, 0.75 wt.% polyaniline, and 1.34 min of evaporation time during the modification process. The predicted responses were 62 LMH·bar^−1^ pure water permeability, 32.4% salt rejection, and 54.95° contact angle. The characterization of the optimized membrane showed improvement in membrane structure, highest membrane surface charge, and better pore distribution as compared to the control.

Recently, the RSM approach has been increasingly utilized in polymeric fields. For example, Hasanzadeh et al. [13] used RSM for multi-objective optimization of the gasification process for waste polystyrene and waste polystyrene foams. The effect of gasification temperature, moisture content, and equivalence ratio was studied and the results showed waste polystyrene foam had a better performance than its counterpart [13]. In another study, RSM was used to study the effect of gasification temperature, moisture content, and equivalence ratio on the air and steam gasification process for polyurethane foam waste [14]. The energy and hydrogen efficiency and gas composition were studied as response variables. The results showed that the air gasification process yields 42.68% and 89.58% hydrogen and energy efficiencies at optimum conditions, respectively, while these values were higher for steam gasification, i.e., 64.02% for hydrogen and 96.52% for energy efficiency [14]. Similarly, other researchers used RSM to optimize printing parameters of sintered 316 L stainless steel [15], manufacturing of small-module plastic gears [16], a system based on biomass-fueled solid oxide fuel cell [17], solid polymer electrolyte-based technique for the electrochemical treatment of wastewater [18], and decolorization of congo red solution using iron-doped zinc oxide nanoparticles [19].

Hence, the overall objective of this research is to investigate the effect of PAA concentration (used as an antiscalant), and UV activation times on the membrane permeation properties using the RSM design of experiments. The optimized membrane will then be tested against model inorganic fouling/mineral scaling solution to ensure its effectiveness. The information from this research will take us one step further toward the development and testing of such multi-functional RO membranes on a pilot scale.

## 2. Materials and Methods

### 2.1. Materials

Thin-film composite polyamide RO membrane (TFC-PA SW30XLE) was acquired from Dow Filmtec/Sterlitech Corp.—Auburn, WA, USA. The acrylic acid (MW 72.06 g/mol) was procured from Sigma Aldrich—Seoul, South Korea. The salts of calcium chloride (CaCl_2_) and sodium chloride (NaCl) were obtained from Scott Science, Kent, UK and sodium sulfate (Na_2_SO_4_) was procured from VWR chemicals—Leuven, Belgium. GO sheets (Code: 763713) were procured from Sigma Aldrich—Saint Louis, MO, USA. The commercially available GO has been characterized in previous studies [7,20]. As per the atomic force microscopy (AFM) technique, the particle size and the thickness of GO are 219 nm and 0.6 nm, respectively. The elemental composition of GO analyzed through the X-ray photoelectron spectroscopy (XPS) technique has shown that it contains 67.8 wt% carbon and 32.2 wt% oxygen [21]. The chemicals required for GO functionalization on the RO membrane are ethylenediamine (ED, BioXtra), HEPES (4-(2-hydroxyethyl)-1-piperazineethanesulfonic acid), MES monohydrate (>99.0%, BioXtra), *N*-(3-Dimethylaminopropyl)-*N*’-ethyl carbodiimide hydrochloride (EDC, 98%) and *N*-hydroxysuccinimide (NHS, 98%). All these chemicals were acquired from Sigma Aldrich—USA.

### 2.2. Surface Modification by UV-Grafting

Thin-film composite (TFC) RO membrane was first thoroughly rinsed to remove preservatives with ultrapure water until the pH and conductivity of the water before rinsing were the same as after rinsing (i.e., at the waste end). The membrane was then stored in deionized (DI) water for 24 h. The GO functionalization of the RO membrane was carried out as reported previously [22,23]. Briefly, the RO membrane was first exposed to the coating solution prepared in MES monohydrate (10 mM) containing EDC (4 mM), NHS (10 mM), and NaCl (0.5 M) for 1 h at pH 5 to convert carboxyl groups on the RO to amine-reactive esters. After 1 h, the excess solution was drained and the membrane was washed twice with de-ionized (DI) water. Then, the membrane was exposed to another coating solution prepared in HEPES (10 mM) containing ED (10 mM), and NaCl (0.15 M) for 0.5 h at pH 7.5. This step was undertaken to form an amide bond between activated esters on the membrane and ED. Meanwhile, GO sheets were dispersed in MES buffer at pH 6, and then probe sonicated for 20 min followed by centrifugation for 30 min. After centrifugation, the supernatant was diluted in MES buffer which was followed by the addition of EDC (2 mM), and NHS (5 mM). The reaction took 15 min which resulted in the conversion of carboxyl groups of GO into amine-reactive esters. Finally, this coating solution was poured on the RO membrane for 1 h at pH 7.2 to form a bond between the amine-reactive esters of the GO with free amine groups of ED on the RO membrane. In the end, the excess solution was removed and the membrane was washed twice with DI water.

The polymerization of acrylic acid was then performed on GO functionalized RO membrane using UV-assisted graft polymerization [24]. Here, the concentration of monomer and the UV radiation time was varied. Previous research has shown that the permeability of the membrane reduces significantly if the concentration of monomer increases to 20 mg·L^−1^ [7]. Therefore, in this research, the concentration of 20 mg·L^−1^ was not exceeded for PAA. Additionally, the irradiation time of 60 min has also proven to be effective for graft polymerization of monomers [8,25]. Other factors that can affect polymerization such as the distance between the lamp and the membrane and UV wavelength were kept constant [26].

To perform graft polymerization, the method reported previously by Homayoonfall et al. [26] was adopted with slight modification. Initially, the membrane was clamped to the glass plate such that the active surface was exposed to the monomer solution and UV radiations. Then, the acrylic acid solution of desired concentration was poured such that its depth over the membrane surface remain constant at any point, i.e.,~0.5 cm. This was followed by irradiation with a UV lamp (Labconco, Kansas, MO, USA, Model G30T8, 254 nm UVC) mounted in a glass chamber (25.7 cm height, 123.2 cm width, and 44.6 cm depth) for a specific time duration. After that, the solution was poured off and the membrane was washed several times with DI water to remove unreacted monomers and homopolymers.

### 2.3. Separation Experiments

**Figure 1:**
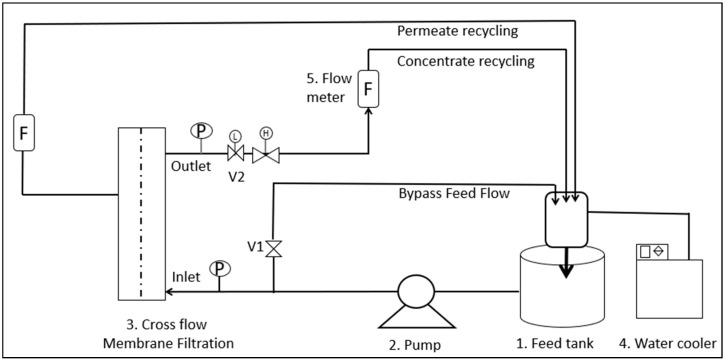
Crossflow filtration setup used for the pure water permeability and scaling tests [8,14].

The pure water permeability (PWP) and salt rejection (%R) were measured for both bared and surface-modified RO membranes. The bench-scale RO setup was prepared for this purpose (Figure 1). Briefly, the feedwater was pumped into the crossflow filtration cell by a hydra-cell pump. The transmembrane pressure (TMP) across the membrane and the flow rate were controlled using the valves located at the feedwater and concentrate water lines. The feedwater temperature was controlled by recirculating the retentate water through the water chiller. The experimental operating conditions were flow rate (1 L·min^−1^), pressure (25 bars), and temperature (25 °C). For PWP, the ultrapure water was used as feedwater, while for %R measurements the sodium chloride (NaCl) solution of 1 g·L^−1^ was used. The PWP and %R were calculated using Equations (1) and (2) [27].

PWP = Q_p_/(∆P·A) (1)

where, Q_p_, A, and ∆P represent permeate water flow rate (L·h^−1^), effective membrane surface area (m^2^), and trans-membrane pressure (bar), respectively.

%R = (C_f_ − C_p_)/C_f_ × 100 (2)

In Equation (2), C_f_ and C_p_ are the conductivity values of feedwater and permeate water, respectively. The conductivity was measured using an electrical conductivity meter (HQ440d, multi, HACH, London, UK).

### 2.4. Inorganic Scaling Studies

To investigate the performance of membranes against mineral scaling, calcium sulfate was used as a model scalant. Briefly, the mixture of sodium sulfate (Na_2_SO_4_) and calcium chloride (CaCl_2_) was used to have the final concentration of 20 mM of calcium and sulfate ions in the feedwater [28]. The ionic strength (IS), pH, and saturation indices with respect to gypsum (SI_gyp_) were calculated using geochemists’ workbench (GWB, V11.0) and were found to be 92.7 mM, 5.66, and −0.13, respectively. The scaling experiments were performed in two steps. Firstly, the membrane compaction was performed at the experimental operating conditions using ultrapure water for 1–2 h until stable permeate flux was obtained. Then, the feedwater was switched to the model scaling solution, and the experiments were continued for 6 h. To ensure the conditions remain constant, the scaling experiments were performed in total recycle mode. At the end of the experiment, the membranes were retrieved and dried. The permeate flux (J) was measured during the time duration of the experiment (Equation (3)) [29] and %R was measured at the end of the experiment (Equation (2)).

J = Q_p_/A (3)

where, Qp represents a permeate flow rate (L·h^−1^), A denotes the active membrane surface area (m^2^), and J is the permeate flux (L·m^−2^·h). The normalized permeate flux (J_N_) was measured using Equation (4) [28].

J_N_ = J/J_o_ (4)

J_o_ represents the initial permeate flux at the start of the experiment and J denotes stabilized permeate flux at a given time.

### 2.5. Membrane Surface Characterization

The membrane surface was characterized using a variety of microscopic and spectroscopic techniques, i.e., scanning electron microscopy (SEM), AFM, Raman microscopy, and attenuated total reflectance—Fourier transform infrared (ATR-FTIR), and XPS. The SEM was done using Nova^TM^ NanoSEM 50 Series (FEI Company, Hillsboro, OR, USA) and the images of the membrane were captured at different magnifications. The AFM was done in conjunction with a nano indenter (AFM-MFP-3D, Asylum Research, Santa Barbara, CA, USA). The average roughness (Ra) and root mean square roughness (RMS) parameters were measured across different locations and the average was calculated. The SEM and AFM techniques were used to investigate the morphological characteristics of the membrane. The Raman microscopy was done to confirm the functionalization of RO membranes using DXR Raman Microscope (Thermo Fisher Scientific, Waltham, MA, USA) with a wavelength of 532 nm, 40 times scanning, and the laser power of 10 mW. The Raman spectra were acquired from 3500 to 1100 cm^−1^. The FTIR spectrum was acquired from 4000 cm^−1^ to 400 cm^−1^ wavenumbers with a nominal spectral resolution of 4 cm^−1^ using the Shimadzu (Kyoto, Japan) FTIR spectrum instrument in a transmittance mode. XPS was done using Axis (Ultra DLD XPS Kratos, Manchester, UK) equipped with a monochromatic Al Kα radiation source (1486.6 eV). The calibration of binding energy was done using carbon (C–C/C–H) at 284.6 eV.

Membrane hydrophilicity was measured using a contact angle instrument (OCA15Pro, Filderstadt, Germany) following the sessile drop method. A volume of 2 µL was dropped on the targeted membrane surface and the contact angle of the droplet with the membrane surface was measured after 5 s using built-in software (SCA20). The measurements were done across various locations and the mean contact angle values are reported.

### 2.6. Study Design

Using RSM, a statistical experimental model was designed that helped to determine the number of experiments required for optimization. This was done to understand the correlation between different parameters of the modification process and their responses. In this research, the correlation between the factors (PAA concentration, and UV radiation time), and the responses (permeate flux, salt rejection) was investigated using RSM.

Within the RSM, the central composite design (CCD) was adopted over Box-Behnken because the former can be utilized when the factors are only two. On the other hand, Box-Behnken requires having at least three factors. Moreover, it does not contain an embedded factorial design and the design points are usually a combination of high factor, low factor, and midpoints. On the other hand, CCD has an embedded factorial design with both central and axial points that can be used to estimate the curvature of a response variable [30]. The central composite inscribed (CCI), a type of CCD, was used to ensure that both the factorial and axial points fall within the desired range of factors being investigated, i.e., PAA concentration (0–60 mg·L^−1^), and UV radiation time (0–60 min). Therefore, the selected low and high values were designated as axial points, and the software computed values for the factorial and central points were as given in Table 1. Design-Expert software (V8.0) was used for this purpose and the software suggested 11 sets of experiments to optimize the modification process.

Experiments were conducted as per the design matrix and the values were recorded and given as input to the software. Design-Expert software used these values to conduct an ANOVA and identified factors that significantly influence the response variables. Accordingly, the response surface plots were generated to visualize the effect of parameters on the process and the conditions were optimized based on the desirability approach of RSM [31].

## 3. Results and Discussion

### 3.1. Effect of Acrylic Acid Concentration

The polymer concentration affects the membrane modification process. At higher concentrations and a higher degree of grafting, the polymer causes resistance to the flow of water through the membrane. Therefore, it is important to investigate the effect of polymer concentration on membrane permeability and salt rejection capabilities. From Table 2, it is evident that the membrane surface modification with PAA increased the permeability from 1.84 (RO bared membrane) to 2.11 LMH·bar^−1^ (at AA concentration of 10 mg·L^−1^). However, at higher concentrations, i.e., at 20 mg·L^−1^, the permeability reduced to 1.90 LMH·bar^−1^. This happened because the polymer density per unit volume of the membrane increased, which further enhanced the membrane resistance to the flow of water through the membrane [32]. Therefore, it is certain that the optimum concentration of AA is between 10 and 20 mg·L^−1^ at which the maximum pure water permeability can be achieved.

### 3.2. Effect of UV Radiation Times

In this research, UV radiations were used to initiate the graft polymerization process. The radiations aid in altering the bonds of the polymer membrane surface and the acrylic acid [24]. However, higher exposure to UV radiations affects the membrane surface, resulting in the ineffective polymerization process. From Table 3, it can be deduced that the membrane permeability is affected by UV radiations for longer durations, i.e., 60 min. Initially, the pure water permeability increased from 1.94 to 2.11 LMH·bar^−1^ with increasing UV radiation time from 0 to 30 min. Nevertheless, after exposure for 60 min, the permeability reduced to 1.93 LMH·bar^−1^. Similarly, salt rejection also reduced significantly from 98% (UV exposure time: 30 min) to 93.5% (UV exposure time: 60 min).

### 3.3. Response Surface Methodology (RSM)

Using RSM, the effect of factors (acrylic acid concentration and UV activation time) was studied on pure water permeability and salt rejection (responses) to obtain the optimized conditions for membrane modification. Table 4 shows the central composite design with different factor values studied and the results of responses obtained. Accordingly, the quadratic models were developed for the studied responses. These models are expressed based on the coded factors and actual factors.

Equations (5) and (6) express the models based on coded factors for permeability and salt rejection (%R), respectively.

Permeability = 2.12 + 0.059A − 0.01B − 0.015AB − 0.15A^2^ − 0.1B^2^ (5)

%R = 98.450 + 0.02A − 1.01B − 0.23AB − 0.012A^2^ − 1.59B^2^ (6)

Equations (7) and (8) express the models based on actual factors for permeability and salt rejection (%R), respectively.

Permeability = 1.514 + 0.071AA concentration + 0.013UV activation time − 0.0001AA concentration × UV activation time − 0.003AA concentration^2^ − 0.0002UV activation time^2^ (7)

%R = 96.032 + 0.096AA concentration + 0.178UV activation time − 0.001AA concentration × UV activation time − 0.002AA concentration^2^ − 0.003UV activation time^2^ (8)

Analysis of variance (ANOVA) was used to verify the model. ANOVA Table summarizes the sum of squares of residuals and regressions, degrees of freedom (df), F-value, *p*-value, and ANOVA coefficients. F-value is a measure of data variance about the mean value. If the F-value is significantly different from unity, it shows that the input variables explain the variation in the mean of the data [33]. Hence, the F-value in Table 5 for the permeability response is 27, which implies that the model is significant. P-value is calculated based on the F-value and df. As the p-value is less than 0.05, i.e., 0.0013 for the model, this shows that the results can be fitted using the quadratic model. In terms of factors, the polymer concentration (*p*-value = 0.0059) has a more significant impact on permeability as compared to UV activation times (*p*-value = 0.4646). Moreover, the interaction between the two factors (AB) has no significant impact on the permeability response, i.e., the *p*-value is 0.4417.

From Table 6, the F-value for the salt rejection response is 21.933, which implies that the model is significant. Furthermore, as the *p*-value is less than 0.05, i.e., 0.0021, this shows that the results can be fitted using the quadratic model. In terms of actual factors, the polymer concentration (*p*-value = 0.908) has an insignificant impact on the salt rejection response, unlike pure water permeability. On the other hand, the UV activation times showed a significant impact on the salt rejection as the p-value value is 0.0016, which is less than 0.05.

The predicted R-square values for permeability and salt rejection responses are not very far away from the Adj. R-square values as mentioned in Table 7. This shows that both models are a good fit, and no transformation of response data is required. Moreover, the value of Adeq Precision indicates a signal-to-noise ratio and it is desirable to have a ratio greater than 4. From Table 7, the ratio of 14.48 for permeability and 13.568 for salt rejection response indicate an adequate signal. Therefore, it can be concluded that both models can be used to navigate the design space.

**Figure 2:**
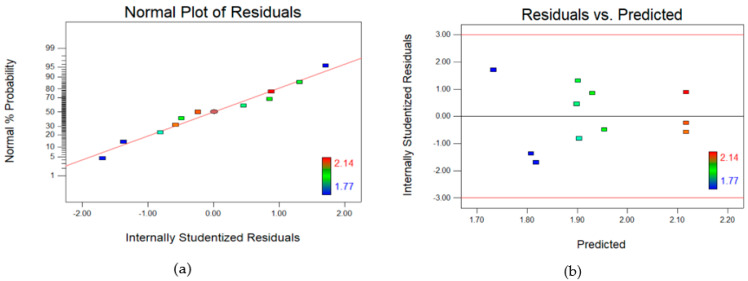

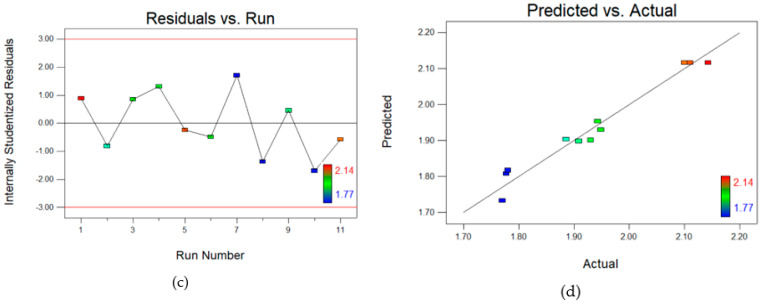
(**a–c**) Residual plots and (**d**) Predicted vs Actual plot for Central composite design (CCD) for pure water permeability.

**Figure 3:**
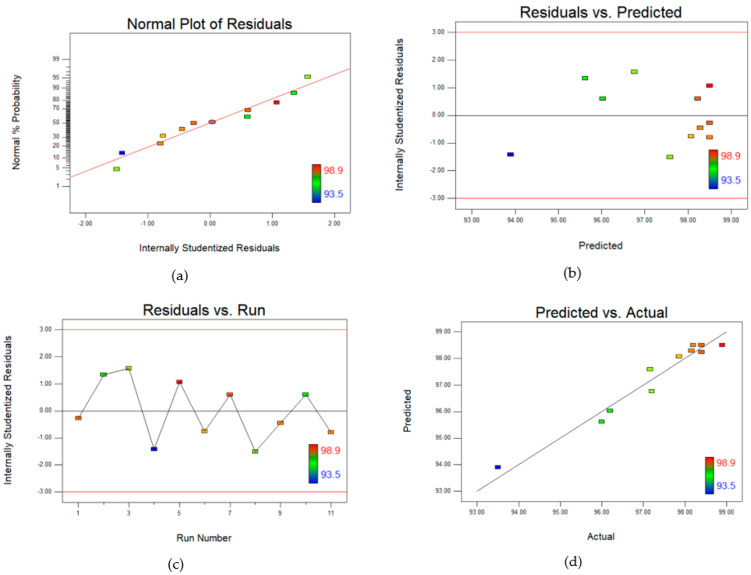
(**a**–**c**) Residual plots and (**d**) Predicted vs Actual plot for Central composite design (CCD) for salt rejection.

To further verify the models, residual diagnostics were used. The residuals are the difference between the responses predicted by the model and the actual responses obtained during the experiments. The results in Figure 2a and Figure 3a show that the residuals for both permeability (Figure 2a) and salt rejection (Figure 3a) closely follow a straight line through the origin in the normal probability plot. This shows that the residuals followed the normal distribution. Hence, no transformation of response data is required for the prediction of results. Moreover, the results in Figure 2b–d and Figure 3b–d also indicate that there were no outliers, and they were distributed within a narrow range.

**Figure 4:**
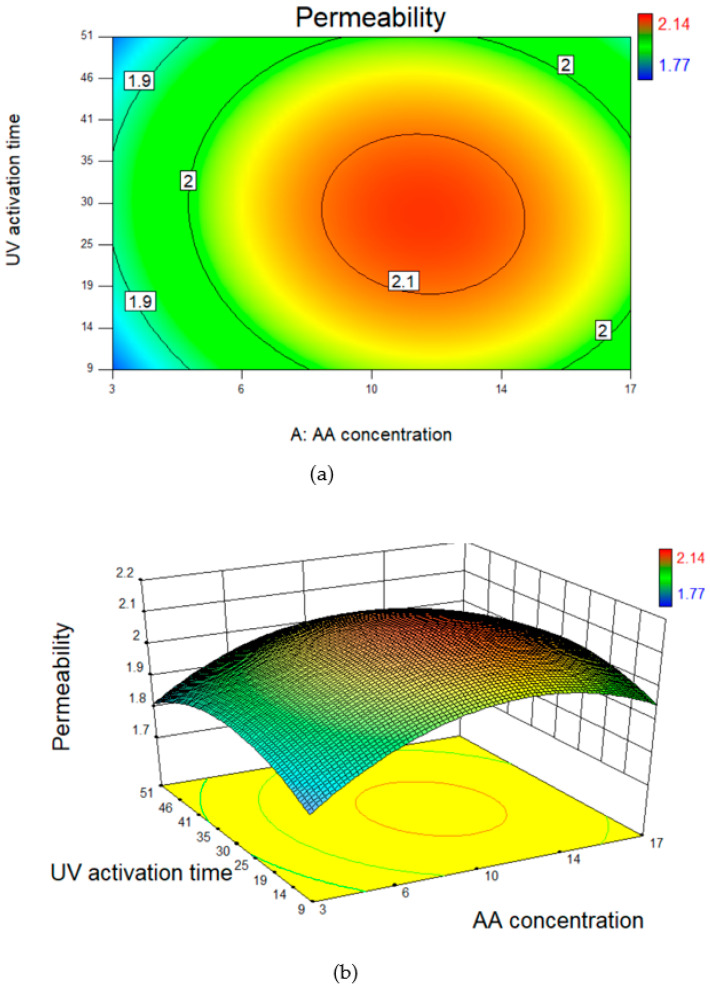
(**a**) Contour line map and (**b**) 3D surface plot for permeability as a function of acrylic acid concentration and UV activation time.

Surface plots were used to further analyze the model parameters. Using these surface plots, one can visualize the effect of individual and combined factors on the response variables. It is done by keeping one factor constant while allowing the effect of change in another factor on the response variable. The contour line map and 3D surface plot in Figure 4 for permeability show that at maximum and minimum polymer concentrations and UV activation times, the permeability was lowest. This indicates that the maximum pure water permeability can be achieved at the mid-range for both polymer concentrations and UV radiation times.

**Figure 5:**
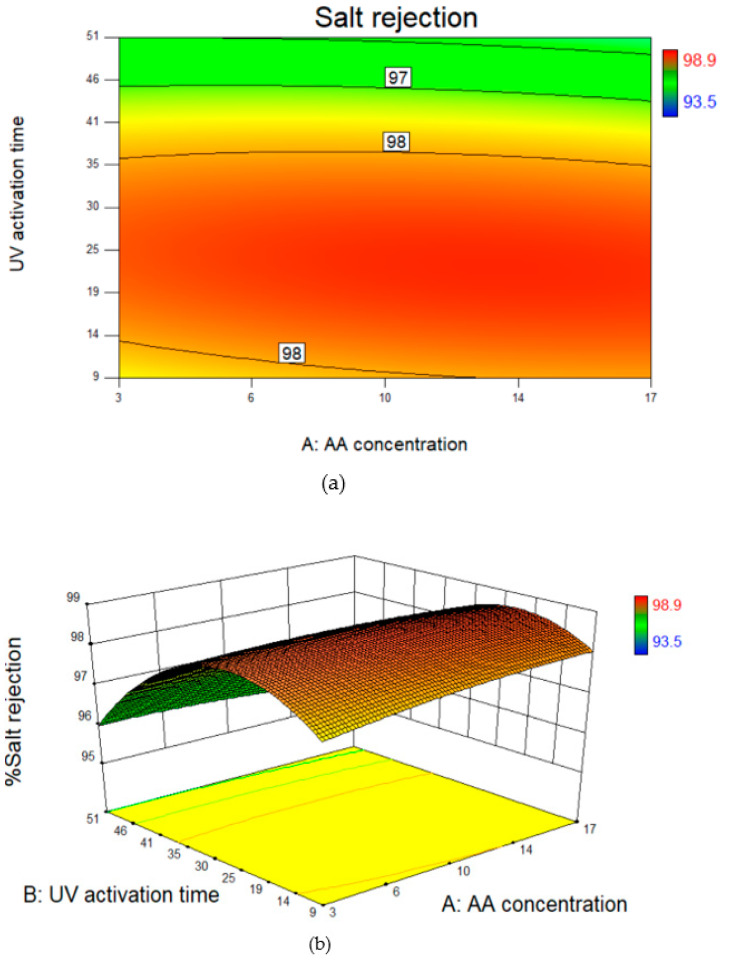
(**a**) Contour line map and (**b**) 3D surface plot for salt rejection as a function of acrylic acid concentration and UV activation time.

In terms of salt rejection, Figure 5 shows that the polymer concentration had little effect on the removal of salts. However, increasing the time of radiation altered the membrane surface causing a reduction in the membrane salt rejection properties.

### 3.4. Process Optimization and Experimental Validation

**Figure 6:**
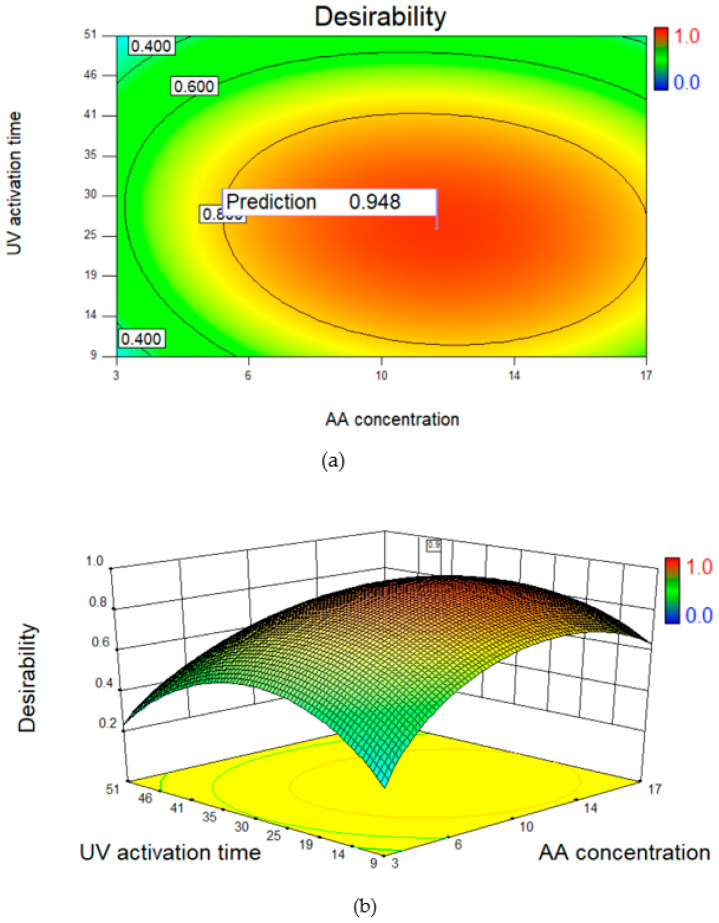
(**a**) Contour plot and (**b**) 3D surface model for the predicted optimized response.

The optimization of independent factors was done to achieve maximum permeability and salt rejection through an optimized response surface model. As per the ANOVA analysis, a quadratic model was plotted for the optimized process (Figure 6). As suggested by the surface plot for the optimized process, the membrane with the best possible performance was predicted. For verification, the predicted parameters were used to modify the membrane surface and the responses were measured for the optimized membrane. The experimental results of the optimized membrane were well in line with the predicted results, confirming the accuracy of the RSM model (Table 8).

### 3.5. Characterization: Property, Morphology, Composition

#### 3.5.1. Membrane Permeation Properties

**Figure 7:**
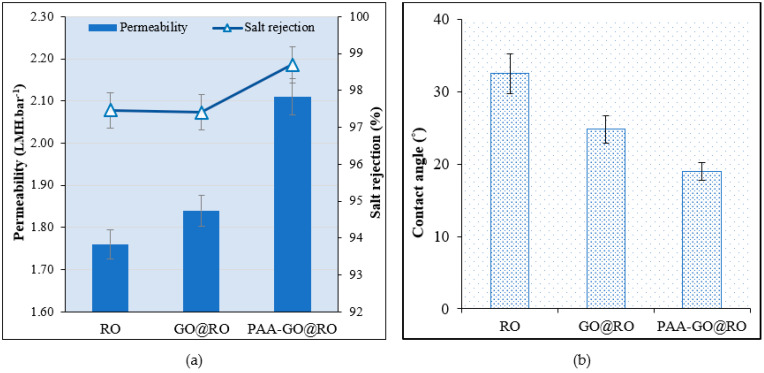
Results of (**a**) Membrane pure water permeability and salt rejection, (**b**) Water contact angle of the bared and optimized modified membrane.

The optimized membrane showed improvement in pure water membrane permeability and salt rejection when sodium chloride was used as a salt solution. There was a more than 15% increase in water permeability (Figure 7a), while the hydrophilicity of the membrane also increased (Figure 7b). This improvement in membrane permeability and hydrophilic properties could be due to the functionalization of hydrophilic polymer on the membrane surface [34]. The presence of carboxyl and hydroxyl functional groups on the hydrophilic polymer also plays a significant role in improving the membrane’s hydrophilic properties [35]. The results showed that the thin layer of the hydrophilic polymer was formed on the membrane surface, which did not hinder the flow of water through the membrane. The increase in both water permeability and salt rejection shows that the modified membranes possessed improved permeation properties.

#### 3.5.2. Membrane Surface Morphology and Functional Group Analysis

**Figure 8:**
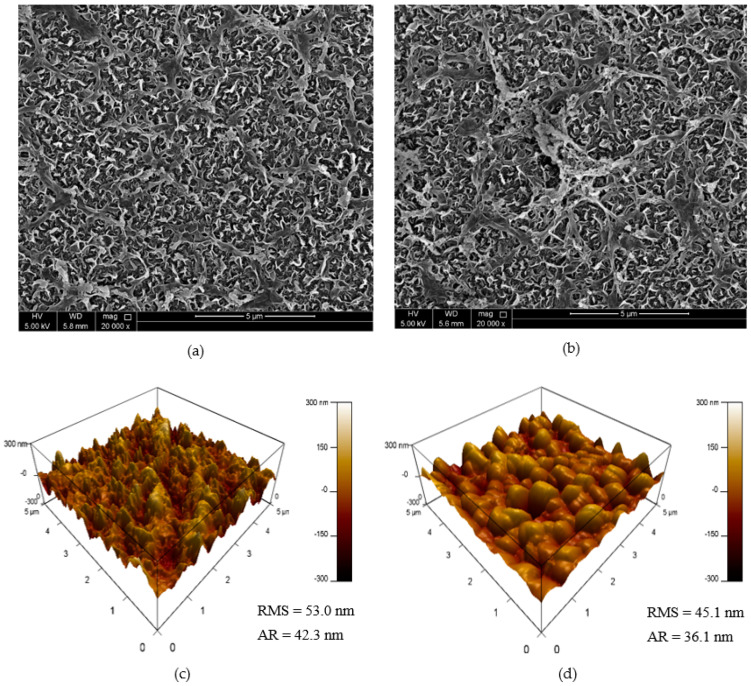
SEM micrographs and AFM images of the membranes (**a**,**c**) RO membrane; (**b**,**d**) PAA-GO@RO optimized membrane.

Membrane surface morphology was studied using microscopic techniques, i.e., SEM and AFM. The ridge and valley structure of the membrane surface, which is typical for polyamide RO membranes, can be seen through SEM micrographs (Figure 8a,b). Although the SEM micrographs did not show significant differences in the morphology, the AFM technique was able to demonstrate a decrease in membrane surface roughness after surface modification (Figure 8c,d). It was noted that the surface roughness reduced from 53.0 nm to 45.1 nm, which is also evident from AFM images through a decrease in valley depths. The results are in line with previous studies in which the improvement in membrane smoothness following surface polymerization has been reported [7].

**Figure 9:**
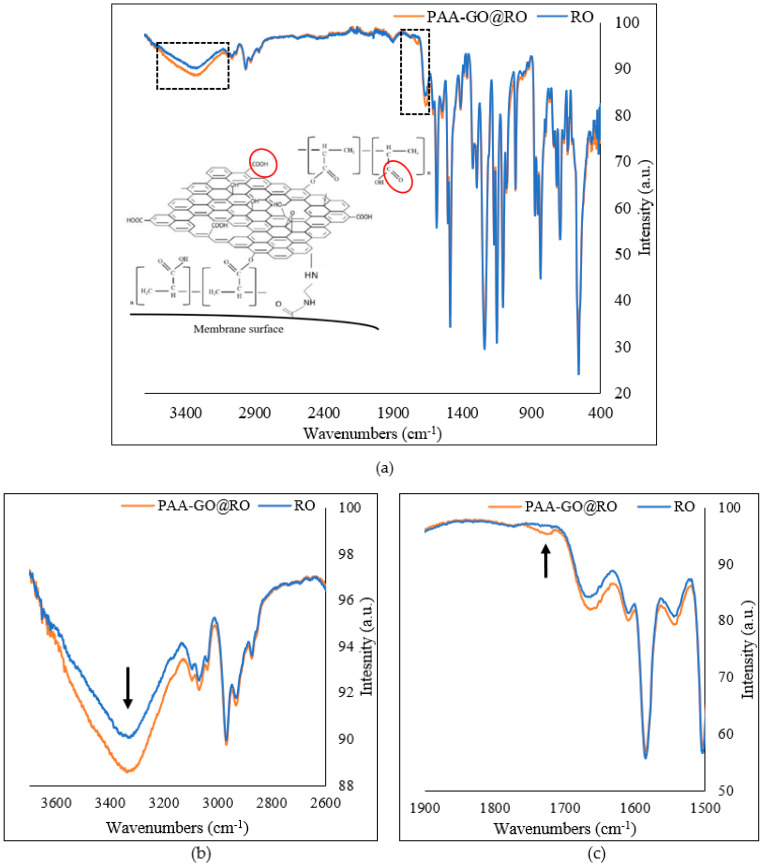
ATR-FTIR spectra of the membranes (**a**) complete spectra 3900–400 cm^−1^; (**b**) spectral range 3900–2900 cm^−1^; (**c**) spectral range 1900–1500 cm^−1^.

The ATR-FTIR spectra of the bared and optimized RO membranes are presented in Figure 9. The functional groups belonging to the polyamide layer of the RO membrane can be seen through 1663 (C=O stretching), 1609 (C=C stretching), and 1541 cm^−1^ (N–H bending) (Figure 9a). After functionalization with GO and PAA, there were no significant differences in the spectra observed as these materials have common functional groups. However, the broad complex band around 3300 cm^−1^ intensified due to the increase in the density of functional groups such as –OH, and C–H groups from GO and polymer (Figure 9b) [36]. Moreover, a new peak appeared at 1720 cm^−1^ confirming the incorporation of the carboxylic acid group (carbonyl, C=O) into the membrane from GO and PAA (Figure 9c) [37].

**Figure 10:**
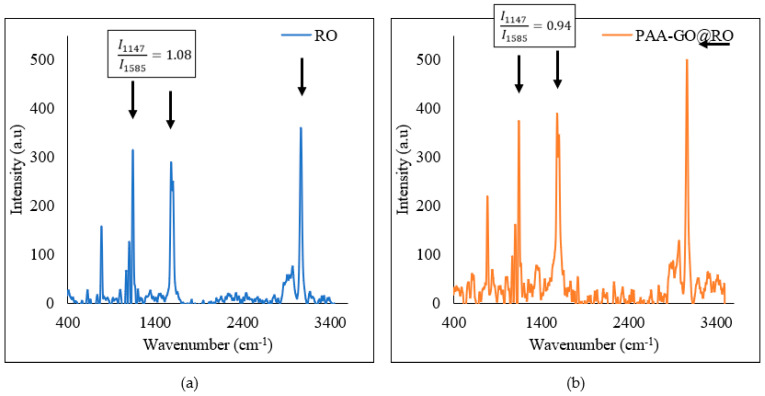
Raman spectra of the membranes (**a**) bared RO membrane; (**b**) PAA-GO@RO optimized membrane.

Figure 10 shows the Raman spectra of the RO and PAA-GO@RO optimized membrane. The difference in the peak intensity ratio of 1147 and 1585 cm^−1^ can be used to demonstrate the difference between the two membranes. It was noted that the ratio decreased from 1.08 for the RO membrane to 0.94 for the PAA-GO@RO membrane. This could be attributed to the presence of the G band from GO at 1590 cm^−1^ [38]. The G band broadens and intensifies the peak at 1585 cm^−1^ originating from C–O–C stretching vibrations of the RO membrane, resulting in a decrease in the ratio of peaks at 1147 and 1585 cm^−1^. Furthermore, the increase in the intensity of the peak at 3027 cm^−1^ showed the increase in the density of hydroxyl groups after GO and polymer functionalization on the RO membrane (Figure 10b).

Table 9 summarizes the results of characterization. The results of the XPS analysis are also presented. The main elements present on the membrane surface were carbon (C), oxygen (O), and nitrogen (N). The XPS results demonstrated the increase in the atomic percentage of oxygen and decrease in the atomic percentage of nitrogen, confirming the functionalization of RO membrane with GO and polymer that resulted in the increase in O/N ratio from 2.2 to 3.2. This increase in oxygen content in the functionalized RO membrane confirmed the increase in abundance of functional groups from PAA and GO such as –OH, C=O, and COOH [8].

### 3.6. Inorganic Fouling Test

**Figure 11:**
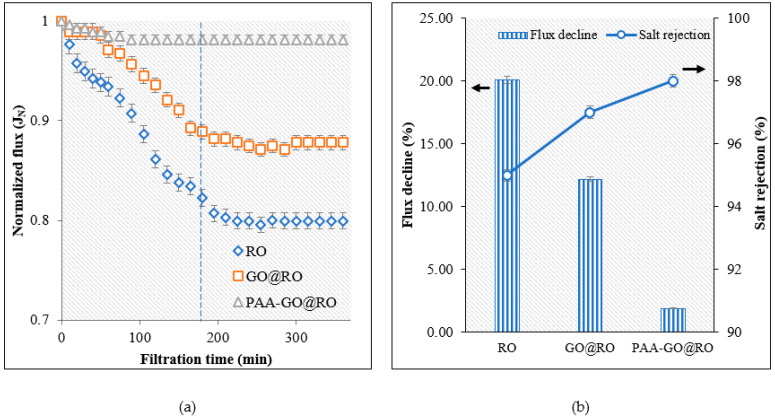
Inorganic fouling tests for the membranes (**a**) Decline of normalized flux with time, (**b**) Comparison of flux decline and salt rejection at the end of experiments.

The optimized membrane was tested for its ability to control mineral scaling. For this purpose, both the bared and optimized RO membrane were exposed to the calcium sulfate solution and a decrease in normalized permeate flux was observed over time. Figure 11 shows that there was a steady decline in permeate flux during the first 180 min, which was followed by the steady-state condition in which no further decline in permeate flux was noted. During this steady-state condition, the rate of attachment of salts to the membrane surface is in equilibrium with the detachment of salts from the membrane surface governed by the flow of water. Overall, the permeate flux for the RO membrane was decreased by 20% by the end of the experiment (Figure 11b). In comparison, the modified RO membrane showed a stable permeate flux with a very slight decrease in flux over time, i.e., ~2% which showed that the inorganic fouling was controlled by the modified membrane. The results also showed that salt rejection for the inorganic scaling solution was maintained at 98% by the modified membrane (Figure 11b).

**Figure 12:**
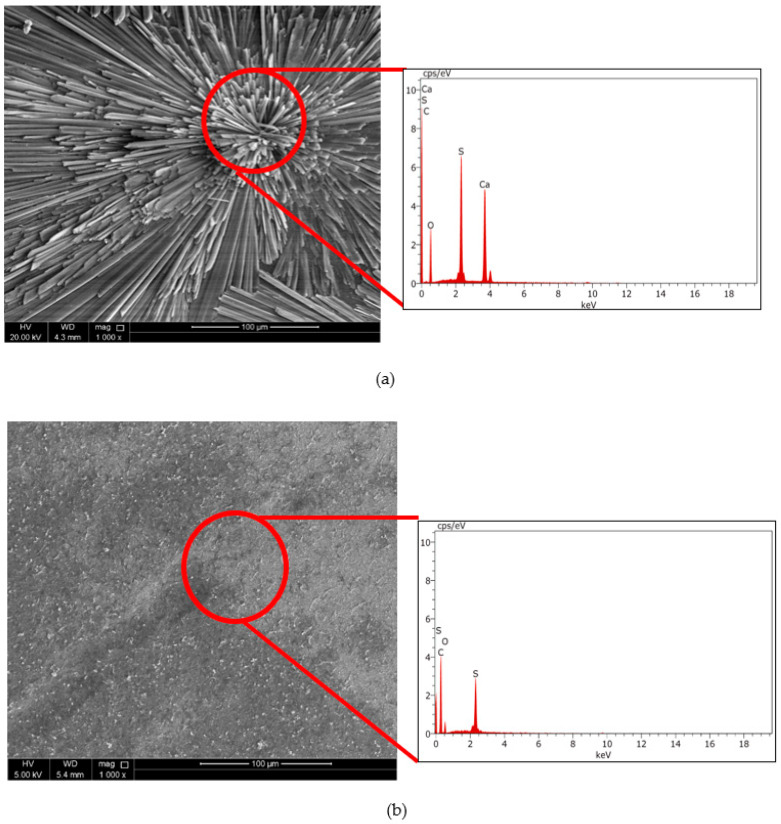
SEM-EDX characterization of the fouling layer (**a**) RO membrane; (**b**) PAA-GO@RO membrane.

The results were further confirmed by using the SEM-EDX technique. After the inorganic fouling experiment, the fouled membranes were dried and subsequently analyzed using the SEM-EDX technique to investigate the formation of scales on the membrane surface. Figure 12a shows the typical needle-like structures of gypsum [39] with EDX spectra confirming the presence of calcium, sulfur, and oxygen atoms. It can be noted that the inorganic fouling resulted due to the surface crystallization in which the crystals originate from the membrane surface, having a core growth region at the surface [22,28]. The SEM micrographs in Figure 12a,b for the bared RO and modified RO membranes, respectively, confirmed that the fouling was significantly controlled in the modified RO membranes. The calcium sulfate crystals were not seen in the modified RO membrane (Figure 12b). The control of inorganic fouling or scaling by the modified RO membranes can be attributed to the ability of polymer antiscalant to inhibit mineral scaling on the membrane [40,41]. Moreover, membrane surface properties also influence membrane fouling [42]. Most of the existing research has shown that the hydrophobic membrane is more prone to membrane fouling, and therefore, hydrophilization of the membrane is recommended to inhibit fouling [37,43]. Similarly, membrane smoothness also helps to prevent membrane fouling by not providing sites for foulant attachment [44]. Hence, the improvement in membrane hydrophilicity and smoothness together with the inherent abilities of polymer antiscalant helped to counter mineral scaling during crossflow filtration tests.

## 4. Conclusions

In this research, RSM was successfully used to optimize the conditions for the preparation of multi-functional polyacrylic acid-graphene oxide (PAA-GO) composite RO membrane. The quadratic models were developed to investigate the effect of AA concentration and UV activation times on pure water permeability and salt rejection. The predicted results from the model were validated by preparing and testing the optimized membrane. The optimum concentration of AA was found to be 11.41 mg·L^−1^ and UV activation time of 28.08 min. The optimized membrane showed improvement in membrane hydrophilicity and surface smoothness. The XPS and FTIR results helped us to understand the improvement in membrane surface composition in terms of carbon and oxygen-containing functional groups.

At the optimized conditions, the prepared membrane showed 98% control of mineral scaling, which was also confirmed by the SEM-EDX technique. The precipitation of calcium sulfate was not found on the modified RO membranes. Therefore, such membranes can be used to control mineral scaling in the water treatment and desalination industries. The successful utilization of these membranes will eliminate the use of antiscalants in the feedwater, which have been proved to contribute to biofouling. These membranes have shown their potential during laboratory-scale studies and their long-term performance testing together with pilot-scale studies will be conducted in the follow-up research. Hence, the outcome of this research will help to further develop such multi-functional RO membranes capable of reducing multiple types of fouling.

## Figures and Tables

**Table 1:** Axial, factorial, and central points studied for different factors.

Factors	Units	Axial Points	Factorial Points	Central Points
PAA concentration	mg·L^−1^	0, 20	3, 17	10
UV radiation time	minutes	0, 60	9, 51	30

**Table 2:** Effects of acrylic acid concentration on permeability and salt rejection.

Run	Factors		Responses	
	AA Concentration	UV Activation Time	Permeability	%R
	mg·L^−1^	Minutes	LMH·bar^−1^	%
0 *	-	-	1.76	97.46
0 **	-	-	1.84	97.41
1	10	30	2.14	98.4
5	10	30	2.11	98.9
7	0	30	1.77	98.4
9	20	30	1.90	98.1
11	10	30	2.10	98.2

0 *—RO bared membrane, 0 **—GO functionalized RO membrane.

**Table 3:** Effect of UV exposure on membrane permeability and salt rejection.

Run	Factors		Responses	
	AA Concentration	UV Activation Time	Permeability	%R
	mg·L^−1^	Minutes	LMH·bar^−1^	%
1	10	30	2.14	98.4
3	10	0	1.94	97.2
4	10	60	1.93	93.5
5	10	30	2.11	98.9
11	10	30	2.1	98.2

**Table 4:** Experimental sets with actual factors in RSM and responses.

Run	Factors		Responses	
	AA Concentration	UV Activation Time	Permeability	%R
	mg·L^−1^	Minutes	LMH·bar^−1^	%
1	10	30	2.14	98.4
2	17	51	1.88	96.0
3	10	0	1.94	97.2
4	10	60	1.93	93.5
5	10	30	2.11	98.9
6	17	9	1.94	97.8
7	0	30	1.77	98.4
8	3	9	1.77	97.1
9	20	30	1.90	98.1
10	3	51	1.78	96.2
11	10	30	2.10	98.2

**Table 5:** ANOVA analysis for the permeability response.

Source	Sum of Squares	df	Mean Square	F-Value	*p*-Value	Contribution (%)
***Model***	0.17	5	0.035	27	0.0013	
AA concentration	0.027	1	0.027	21.1	0.0059	12.18
UV activation time	0.0008	1	0.0008	0.63	0.4646	0.36
AA concentration × UV activation time	0.0009	1	0.0009	0.70	0.4417	0.41
AA concentration^2^	0.13	1	0.13	98.1	0.0002	58.64
UV activation time^2^	0.057	1	0.057	43.8	0.0012	25.71
Residual	0.006	5	0.001			
Total	0.2217	10				
Lack of Fit	0.005	3	0.001	3.61	0.2244	

**Table 6:** ANOVA analysis for the salt rejection response.

Source	Sum of Squares	Df	Mean Square	F-Value	*p*-Value	Contribution (%)
***Model***	23.148	5	4.629	21.933	0.0021	
AA concentration	0.003	1	0.003	0.014	0.9089	0.01
UV activation time	8.129	1	8.129	38.516	0.0016	34.61
AA concentration × UV activation time	0.202	1	0.202	0.959	0.3723	0.86
AA concentration^2^	0.081	1	0.081	0.384	0.5625	0.34
UV activation time^2^	14.017	1	14.017	66.408	0.0005	59.68
Residual	1.055	5	0.211			
Total	23.487	10				
Lack of Fit	0.795	3	0.265	2.039416	0.3457	

**Table 7:** The R-squared parameters for the permeability and salt rejection response models.

	Permeability	Salt Rejection
**R-Squared**	0.964	0.956
**Adj R-Squared**	0.928	0.912
**Pred R-Squared**	0.773	0.742
**Adeq Precision**	14.48	13.568

**Table 8:** Predicted and experimental responses at optimum conditions.

Membrane	Optimized Conditions		RSM Prediction		Desirability	Experimental	
	AA Concentration (mg·L^−1^)	UV Activation Time (min.)	Permeability (LMH·bar^−1^)	% R (%)		Permeability (LMH·bar^−1^)	% R (%)
PAA-GO@RO	11.48	25.66	2.12115	98.6	0.948	2.11	98.7

**Table 9:** Comparison of membrane permeation properties, and surface characterization (XPS, AFM, Raman) before and after functionalization.

	Permeability	Salt Rejection	Elemental Composition				Membrane Surface Roughness Parameters		Raman Spectrum
	(LMH·bar^−1^)	% R	C (283 eV)	O (530 eV)	N (398 eV)	O/N	RMS	AR	I1147/I1585
RO	1.7 ± 0.1	97.4 ± 0.1	74.12	17.55	7.96	2.20	53.0	42.3	1.08
PAA-GO@RO (optimized)	2.1 ± 0.1	98.7 ± 0.1	73.74	24.41	7.39	3.30	45.1	36.1	0.94

## Data Availability

Not applicable.

## References

[1] Bahar O.U. (2022). Membrane autopsy study to characterize fouling type of RO membrane used in an industrial zone wastewater reuse plant. Desalination.

[2] Wang J., Li S., Guan Y., Zhu C., Gong G., Hu Y. (2022). Novel RO membranes fabricated by grafting sulfonamide group: Improving water permeability, fouling resistance and chlorine resistant performance. J. Membr. Sci..

[3] Park H.M., Yoo J., Lee Y.T. (2019). Improved fouling resistance for RO membranes by a surface modification method. J. Ind. Eng. Chem..

[4] Gu Q.A., Liu L., Wang Y., Yu C. (2021). Surface modification of polyamide reverse osmosis membranes with small-molecule zwitterions for enhanced fouling resistance: A molecular simulation study. Phys. Chem. Chem. Phys..

[5] Saleem H., Zaidi S.J. (2020). Nanoparticles in reverse osmosis membranes for desalination: A state-of-the-art review. Desalination.

[6] Safarpour M., Khataee A., Vatanpour V. (2015). Thin-film nanocomposite reverse osmosis membrane modified by reduced graphene oxide/TiO2 with improved desalination performance. J. Membr. Sci..

[7] Ashfaq M.Y., Al-Ghouti M.A., Zouari N. (2020). Functionalization of reverse osmosis membrane with graphene oxide and polyacrylic acid to control biofouling and mineral scaling. Sci. Total Environ..

[8] Ansari A., Pena-Bahamonde J., Wang M., Devin L., Shaffer D.L., Hu Y., Rodrigues D.F. (2021). Polyacrylic acid-brushes tethered to graphene oxide membrane coating for scaling and biofouling mitigation on reverse osmosis membranes. J. Membr. Sci..

[9] Baih M.A., Saffaj H., Aziz K., Bakka A., El Baraka N., Zidouh H., Mamouni R., Saffaj N. (2021). Statistical optimization of the elaboration of ceramic membrane support using Plackett-Burman and response surface methodology. Mater. Today Proc..

[10] Yan X., Wang G., Ma C., Li J., Cheng S., Yang C., Chen L. (2021). Effects of pollutants in alkali/surfactant/polymer (ASP) flooding oilfield wastewater on membrane fouling in direct contact membrane distillation by response surface methodology. Chemosphere.

[11] Chung Y.T., Ng L.Y., Mohammad A.W. (2014). Sulfonated-polysulfone membrane surface modification by employing methacrylic acid through UV-grafting: Optimization through response surface methodology approach. J. Ind. Eng. Chem..

[12] Razali N.F., Mohammad A.W., Hilal N., Leo C.P., Alam J. (2013). Optimisation of polyethersulfone/polyaniline blended membranes using response surface methodology approach. Desalination.

[13] Hasanzadeh R., Azdast T., Mojaver M., Park C.B. (2022). High-efficiency and low-pollutant waste polystyrene and waste polystyrene foam gasification: Comprehensive comparison analysis, multi-objective optimization and multi-criteria decision analysis. Fuel.

[14] Hasanzadeh R., Mojaver M., Azdast T., Park C.B. (2022). A novel systematic multi-objective optimization to achieve high-efficiency and low-emission waste polymeric foam gasification using response surface methodology and TOPSIS method. Chem. Eng. J..

[15] Tosto C., Tirillò J., Sarasini F., Sergi C., Cicala G. (2022). Fused Deposition Modeling Parameter Optimization for Cost-Effective Metal Part Printing. Polymers.

[16] Wu W., He X., Li B., Shan Z. (2022). An Effective Shrinkage Control Method for Tooth Profile Accuracy Improvement of Micro-Injection-Molded Small-Module Plastic Gears. Polymers.

[17] Mojaver P., Khalilarya S., Chitsaz A. (2020). Multi-objective optimization and decision analysis of a system based on biomass fueled SOFC using couple method of entropy/VIKOR. Energy Convers. Manag..

[18] Abidi J., Clematis D., Samet Y., Delucchi M., Cademartori D., Panizza M. (2022). Influence of anode material and chlorides in the new-gen solid polymer electrolyte cell for electrochemical oxidation–Optimization of Chloroxylenol degradation with response surface methodology. J. Electroanal. Chem..

[19] Bhavani Y., Babu N.C., Kumar K.U. Decolorisation of Congo red synthetic solution using Fe doped ZnO nano particles and optimization using response surface methodology. Mater. Today Proc..

[20] Mendonça M.C.P., Rodrigues N.P., de Jesus M.P., Amorim M.J.B. (2019). Graphene-based nanomaterials in soil: Ecotoxicity assessment using Enchytraeus crypticus reduced full life cycle. Nanomaterials.

[21] Clemente Z., Silva G.H., Nunes M.C.S., Martinez D.S.T., Maurer-Morelli C.V., Thomaz A.A., Castro V.L.S. (2019). Exploring the mechanisms of graphene oxide behavioral and morphological changes in zebrafish. Environ. Sci. Pollut. Res..

[22] Ashfaq M.Y., Al-Ghouti M.A., Zouari N. (2020). Functionalization of reverse osmosis membrane with graphene oxide to reduce both membrane scaling and biofouling. Carbon.

[23] Perreault F., Tousley M.E., Elimelech M. (2014). Thin-film composite polyamide membranes functionalized with biocidal graphene oxide nanosheets. Environ. Sci. Technol. Lett..

[24] Suresh D., Goh P.S., Ismail A.F., Hilal N. (2021). Surface Design of Liquid Separation Membrane through Graft Polymerization: A State of the Art Review. Membranes.

[25] Homayoonfal M., Akbari A., Mehrnia M.R. (2010). Preparation of polysulfone nanofiltration membranes by UV-assisted grafting polymerization for water softening. Desalination.

[26] Asadollahi M., Bastani D., Mousavi S.A., Heydari H., Mousavi D.V. (2020). Improvement of performance and fouling resistance of polyamide reverse osmosis membranes using acrylamide and TiO_2_ nanoparticles under UV irradiation for water desalination. J. Appl. Polym. Sci..

[27] Alkhouzaam A., Qiblawey H. (2021). Synergetic effects of dodecylamine-functionalized graphene oxide nanoparticles on antifouling and antibacterial properties of polysulfone ultrafiltration membranes. J. Water Proc. Eng..

[28] Ashfaq M.Y., Al-Ghouti M.A., Da’na D.A., Qiblawey H., Zouari N. (2020). Effect of concentration of calcium and sulfate ions on gypsum scaling of reverse osmosis membrane, mechanistic study. J. Mat. Res. Technol..

[29] Ashfaq M.Y., Al-Ghouti M.A., Da’na D.A., Qiblawey H., Zouari N. (2020). Investigating the effect of temperature on calcium sulfate scaling of reverse osmosis membranes using FTIR, SEM-EDX and multivariate analysis. Sci. Total Environ..

[30] National Institute of Standards and Technology (NIST) U.S. Department of Commerce e-Handbook of Statistical Methods. http://www.itl.nist.gov/div898/handbook.

[31] Chelladurai S.J.S., Murugan K., Ray A.P., Upadhyaya M., Narasimharaj V., Gnanasekaran S. (2021). Optimization of process parameters using response surface methodology: A review. Mater. Today Proc..

[32] Yi S., Su Y., Qi B., Su Z., Wan Y. (2010). Application of response surface methodology and central composite rotatable design in optimizing the preparation conditions of vinyltriethoxysilane modified silicalite/polydimethylsiloxane hybrid pervaporation membranes. Sep. Purif. Technol..

[33] Khayet M., Cojocaru C., Essalhi M. (2011). Artificial neural network modeling and response surface methodology desalination by reverse osmosis. J. Membr. Sci..

[34] Mansourpanah Y., Kakanejadifard A., Dehrizi F.G., Tabatabaei M., Afarani H.S. (2015). Increasing and enhancing the performance and antifouling characteristics of PES membranes using acrylic acid and microwave-modified chitosan. Korean J. Chem. Eng..

[35] Ng Z.C., Lau W.J., Lai G.S., Meng J., Gao H., Ismail A.F. (2022). Facile fabrication of polyethyleneimine interlayer-assisted graphene oxide incorporated reverse osmosis membranes for water desalination. Desalination.

[36] Mansourpanah Y., Afarani H.S., Alizadeh K., Tabatabaei M. (2013). Enhancing the performance and antifouling properties of nanoporous PES membranes using microwave-assisted grafting of chitosan. Desalination.

[37] Ngo T.H.A., Do K.D., Tran D.T. (2017). Surface modification of polyamide TFC membranes via redox-initiated graft polymerization of acrylic acid. J. Appl. Polym. Sci..

[38] Nguyen T.T.D., Nguyen D., Doan N.H., Phu P.V., Huynh V.T., Hoang V.H., Phan T.B., Kinashi K., Nguyen P.T. (2022). In-depth understanding of the photoreduction of graphene oxide to reduced-graphene oxide on TiO_2_ surface: Statistical analysis of X-ray photoelectron and Raman spectroscopy data. Appl. Surf. Sci..

[39] Melliti E., Bruggen B.V., Elfil H. (2022). Combined iron oxides and gypsum fouling of Reverse Osmosis membranes during desalination process. J. Membr. Sci..

[40] Yu W., Song D., Chen W., Yang H. (2020). Antiscalants in RO membrane scaling control. Wat. Res..

[41] Tong T., Wallace A.F., Zhao S., Wang Z. (2019). Mineral scaling in membrane desalination: Mechanisms, mitigation strategies, and feasibility of scaling-resistant membranes. J. Membr. Sci..

[42] Saini B., Sinha M.K., Dey A. (2022). Functionalized polymeric smart membrane for remediation of emerging environmental contaminants from industrial sources: Synthesis, characterization and potential applications. Process. Saf. Environ. Prot..

[43] Zhang C., Bao Q., Wu H., Shao M., Wang X., Xu Q. (2022). Impact of polysaccharide and protein interactions on membrane fouling: Particle deposition and layer formation. Chemosphere.

[44] Jiang S., Li Y., Ladewig B.P. (2017). A review of reverse osmosis membrane fouling and control strategies. Sci. Total Environ..

